# Gluteal pyomyositis in a non-tropical region as a rare cause of sciatic nerve compression: a case report

**DOI:** 10.1186/1752-1947-2-204

**Published:** 2008-06-12

**Authors:** Tamer Kamal, Mathew Hall, Ashraf Moharam, Michael Sharr, Jonathan Walczak

**Affiliations:** 1 Orthopaedic and Traumatology Department, Princess Royal University Hospital, Orpington, Kent, UK; 2 Orthopaedic and Traumatology Department, Cairo University Hospital, Cairo, Egypt

## Abstract

**Introduction:**

Pyomyositis, or isolated abscess formation within a skeletal muscle, is a relatively common condition in tropical climates but it is only encountered rarely in temperate zones.

**Case presentation:**

We present a case of non-tropical pyomyositis of the gluteal muscle in a 26-year-old, previously healthy man from the United Kingdom, who initially presented with sciatica-like symptoms which began 3 days after a mosquito bite on his nose, which had become infected and discharged pus.

**Conclusion:**

Gluteal pyomyositis involving the sciatic nerve may initially present as radiculopathy. Mosquito bites may have been the source of transient bacteraemia that contributed to muscle suppuration in this patient. This may explain, at least in part, the increased incidence of pyomyositis in healthy individuals living in tropical regions.

## Introduction

Pyomyositis in tropical regions often occurs in healthy young people and is thought to result from coincident transient bacteraemia and minor muscle trauma [1]. In non-tropical regions pyomyositis arises primarily in patients with compromised immunity. Non-tropical pyomyositis in healthy individuals is extremely rare, with only a few case reports since its first description in 1971 [1]. Among the reasons suggested for the demographic distribution of this disease are the greater incidence of immunodeficiency, malnutrition and viral infection observed in tropical regions [2-4].

## Case presentation

Following a game of volleyball whilst on holiday in Spain, a healthy, athletic 26-year-old man of Caucasian origin from the United Kingdom developed a pain in the posterior region of his left thigh and buttock. The only recent medical history of note was a mosquito bite on the nose, which had become infected and discharged pus 3 days earlier. At a hospital in Spain, the pain was attributed to a radiculopathic process and managed with bed rest and non-steroidal and opiate analgesia. Despite this the symptoms worsened, with increasing pain and malaise and, eventually, he developed a noticeable limp.

Upon his return to the United Kingdom, the patient was re-assessed by a consultant neurosurgeon. At this time, the patient appeared mildly unwell, with the only physical finding a limited ability to raise his left leg. The patient was initially diagnosed with left sided sciatica, likely resulting from a prolapsed intervertebral disc. Magnetic resonance imaging (MRI) of the lumbo-sacral spine, however, revealed no causative pathology, and the patient was referred to a consultant orthopaedic surgeon for further investigation.

**Figure 1:**
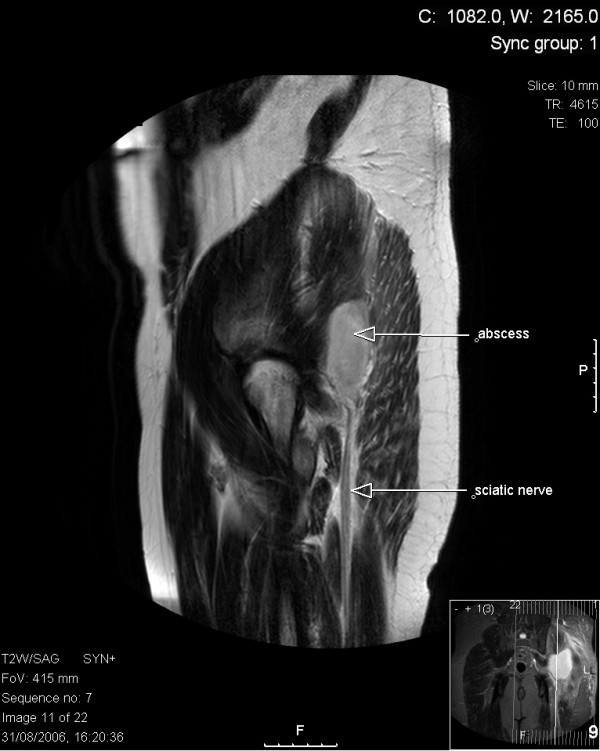
Axial T2 magnetic resonance imaging section through the hip region showing abscess collection in relation to the left sciatic nerve.

**Figure 2:**
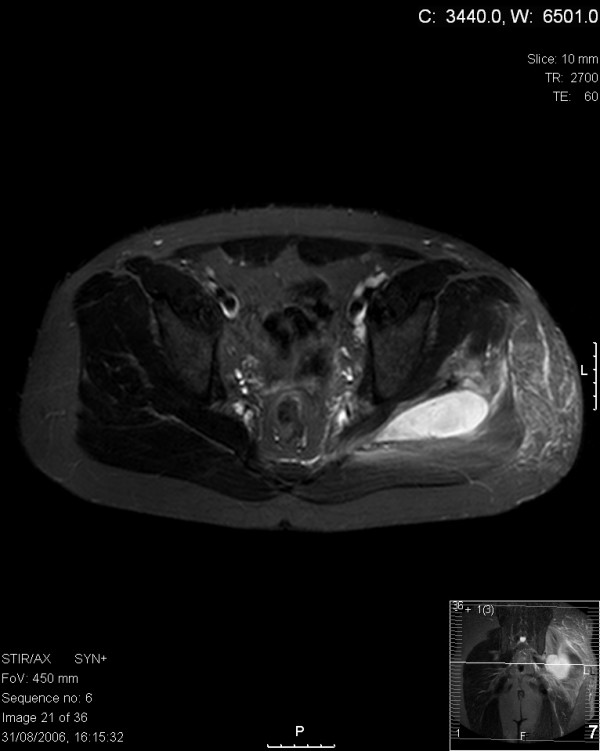
Transverse T2 magnetic resonance imaging section through the hip region showing abscess collection in relation to the sciatic nerve.

The following day, the patient became systemically unwell, in that he became pale, sweaty and tachycardic with low-grade pyrexia. Passive left hip movements were extremely painful, and a palpable area of warmth and induration over the left gluteal region was now evident. Haematological and serological investigation revealed a total leukocyte count of 18.4 (14.0 neutrophils), C-reactive protein of 242 and an erythrocyte sedimentation rate of 40 mm/hour. MRI of the hip and buttock area showed a collection of fluid posterior to the femur, between the gluteus maximus and medius muscles, and pressing upon the sciatic nerve (Figures 1 and 2).

Later that day, the affected area was explored surgically through a posterior approach and 30 ml of purulent material was drained, followed by a thorough washout of the cavity. The patient was treated with parental flucloxacillin for 3 days (1 g four times a day), and he was discharged on the fourth postoperative day fully able to bear weight on his left leg. He made a full and uneventful recovery following treatment with oral flucloxacillin for 4 weeks (1 g four times a day). *Staphylococcus aureus *sensitive to flucloxacillin was cultured from the abscess fluid.

## Discussion

The pathogenesis of pyomyositis is thought to involve two distinct but coincident events: muscle injury, either acute or due to overuse, giving rise to a sub-clinical intramuscular haematoma; and bacteraemia occurring within a few days of the muscle trauma and presumably seeding the haematoma with organisms. In the United States, bacterial pyomyositis in children and young adults has been found to occur after arm wrestling, playing volleyball or swimming [5-7], with the most frequent anatomical locations being the thighs, shoulders, calves and paravertebral regions. Most cases of pyomyositis in both tropical and temperate regions are caused by *S. aureus *[8], although it may also be caused by other organisms, including *S. epidermidis, Streptococci*, and Gram-negative organisms such as *Escherichia coli*, *Klebsiella *and *Pseudomonas *species. These causative organisms may enter through skin lesions, abrasions, pustules or open or penetrating wounds.

Clinical pyomyositis can develop slowly, with its pathogenesis divided into three phases. Initially, cramps or aches develop in the affected area, accompanied by mild constitutional symptoms. The second or suppurative phase consists of clear signs of local infection and/or inflammation and progressive systemic illness; this phase may take up to 3 weeks to develop fully, and aspiration during this phase may yield purulent material. If untreated, this may lead to the third phase, which is characterized by high fevers, excruciating pain, signs of toxicity and even septic shock [9].

This report illustrates the difficulty in correctly diagnosing a rare pathology that initially presents with common symptoms. This patient initially presented with symptoms and signs mimicking sciatica secondary to a prolapsed inter-vertebral disc. The true suppurative aetiology of this case of non-tropical pyomyositis became evident only after the development of clinical evidence of local and systemic infection (that is, progression from phase one to two). Since pyomyositis may arise in any skeletal muscle, the earliest evidence of the disease may arise from symptoms caused by the occupation of space by the fluid collection. In this patient, the fluid collection caused sciatic nerve compression.

In this patient, it is likely that the mosquito bite on the nose led to bacteraemia and the seeding of *Staphylococci *into a subclinical gluteal muscle haematoma sustained from exertion while playing volleyball. While physical exertion and minor muscle trauma are common events in young people from both tropical and non-tropical climates, biting insects, particularly mosquitoes, are more prevalent in tropical regions. Insect bites (infected or otherwise) may therefore generate a source of transient bacteraemia in tropical regions, increasing the likelihood of seeding into damaged skeletal muscle.

## Conclusion

Pyomyositis should be part of the differential diagnosis in any patient with a clinical abnormality arising from compression or compromise of any structures related to skeletal muscles. The presence of even mild systemic illness should increase suspicion of this disease. Non-tropical pyomyositis in a healthy young person is a rare event. The association of this condition with an infected mosquito bite suggests that insect bites may play a causative role in the much more common but demographically distinct disease, tropical pyomyositis.

## Competing interests

The authors declare that they have no competing interests.

## Consent

Written informed consent was obtained from the patient for publication of this case report and any accompanying images. A copy of the written consent is available for review by the Editor-in-Chief of this journal.

## Authors' contributions

TK undertook writing and the literature review and submitted the article, MH undertook the literature search and manuscript preparation, AM contributed to the writing and literature review, JW and MS were responsible for diagnosis, patient management and review. All authors read and approved the final manuscript.
